# Stroke in Sierra Leone. the stroke risk factors for people with HIV: A prospective case-control study

**DOI:** 10.1016/j.jstrokecerebrovasdis.2023.107279

**Published:** 2023-07-29

**Authors:** Mamadu Baldeh, Daniel Youkee, Sulaiman Lakoh, Anthony Rudd, Peter Langhorne, Gibrilla F Deen, Zainab F Conteh, Durodami R Lisk, Jessica O’Hara, Melvina Thompson, Michael Tanu Brima, Yanzhong Wang, Charles DA Wolfe, Catherine M Sackley

**Affiliations:** a College of Medicine and Allied Health Sciences, University of Sierra Leone, Freetown, Sierra Leone; b Medical Research Council Gambia at London School of Hygiene and Tropical Medicine, UK; c School of Life Course and Population Sciences, King’s College London, London, UK; d Connaught Teaching Hospital, University of Sierra Leone teaching Hospital Complex, Freetown, Sierra Leone; e Academic Section of Geriatric Medicine, School of Cardiovascular and Metabolic Health, University of Glasgow; f National Institute for Health Research Collaboration for Leadership in Applied Health Research and Care South London, London, UK; g Faculty of Medicine and Health Sciences, University of Nottingham, UK

**Keywords:** Stroke, Sierra Leone, Sub – Saharan Africa, HIV, NIHSS, Barthel index, Outcome

## Abstract

**Background:**

HIV infection rates are relatively low in Sierra Leone and in West Africa but the contribution of HIV to the risk factors for stroke and outcomes is unknown. In this study, we examined stroke types, presentation, risk factors and outcome in HIV stroke patients compared with controls.

**Methods:**

We used data from the Stroke in Sierra Leone Study at 2 tertiary hospitals in Freetown, Sierra Leone. A case control design was used to compare stroke type, presentation, risk factors and outcome in sero-positive HIV patients with HIV negative stroke controls. Controls were matched for age and gender and a 1:4 ratio cases to controls was used to optimize power. Analysis was performed using the Pearson x^2^ for categorical variable, Paired-T test and Mann-Whitney U test for continuous variables. A p-value of less than 0.05 was taken as the level of statistical significance.

**Results:**

Of 511 (51.8%) stroke patients tested for HIV, 36 (7.1%) were positive. Univariate unmatched analysis showed a stroke mean age of 49 years in HIV-positive versus 58 years in HIV-negative population (p = <0.001). In the case-control group, ischaemic stroke is the major type reported in both populations, HIV-negative population: 77 (53.5%) versus HIV-positive: 25 (69.4%) (p = 0.084). Hypertension is the most prevalent risk factor in both groups, HIV-positive: 23 (63.9%) versus HIV-negative: 409 (86.1%) (p = 0.001). Lower CD4+ count is associated in-hospital mortality (p = <0.001).

**Conclusion:**

These findings support the current call for timely management of stroke and HIV through integrated care.

## Introduction

Stroke is a leading and rapidly increasing cause of adult deaths and disability in Sub-Saharan Africa (SSA)^1,2^. The human immunodeficiency virus (HIV) infection is a leading cause of death and disability in Africa particularly among the 25–49-year age group. The evidence for HIV as a risk factor for vascular disease has been demonstrated in a systematic review of over 300,000 HIV sero-positive individuals^3^.

With the advent of highly active antiretroviral therapy (HAART), HIV infection has become a chronic disease with a longer life expectancy in People Living with HIV (PLHIV). The increase in survival of PLHIV over the past few years has also contributed to a 10-fold increase in the burden of non-communicable diseases (NCDs) in this population^4,5^. However, recent studies in Ghana and Malawi showed that in the first 6-months of initiation of HAART, PLHIV were associated with a higher risk of stroke occurrence compared to untreated HIV patients. This was attributed to immunosuppression and Immune Reconstitution Inflammatory Syndrome (IRIS) processes as a result of HAART use^6,7^.

Globally, the burden of non-communicable diseases such as stroke in PLHIV is expected to continue to rise to unprecedented levels, partly due to lifestyle changes, longer survival, and metabolic abnormalities related to antiretroviral therapy^5^. In a recent study in Malawi, PLHIV aged 45 years or younger were at high risk of stroke. Traditional risk factors for stroke, such as smoking, hypertension, and diabetes, can combine as a causal link with direct HIV infection, resulting in an almost 50% increased burden of stroke in PLHIV^6^. Metabolic syndrome among PLHIV was reported at 21.5% compared to 12% of uninfected patients in a recent systemic review of multiple cross-sectional studies comprising of about 500 HIV uninfected and 700 HIV infected participants in SSA^8^.

Sierra Leone has a low HIV prevalence, with a national sero-prevalence of 1.7% ^9^. However, despite these encouraging national statistics, less than 50% of PLHIV are receiving HAART^10^. As a result, many PLHIV in Sierra Leone are at greater risk of developing advanced HIV disease and therefore are also susceptible to stroke from untreated HIV infection compared to the risk attributed to early initiation of HAART^10,11,12^. The burden of both stroke and HIV in our study setting, Connaught Hospital, the adult tertiary referral hospital for Sierra Leone is high, reinforcing the need to know more about stroke in PLHIV to inform policy and practice^12–14^.

The Stroke in Sierra Leone (SISLE) register^14^ was established in 2019 to determine the prevalence of stroke risk factors, stroke phenotypes, patient pathways to care, quality of hospital care and outcomes after stroke. In this case control study, we aim to examine the factors associated with stroke among PLHIV and compare traditional risk factors and clinical presentation with a gender and age-matched sample of stroke patients without HIV at Connaught Hospital in Freetown, Sierra Leone.

## Methods

### Study site

The Stroke in Sierra Leone (SISLE) stroke register is a prospective hospital-based stroke register in Sierra Leone’s two main adult referral hospitals in Freetown [methods described in 14.]. These facilities act as tertiary hubs for both stroke care and HIV, serving over 1.2 million inhabitants in the Western Area and nationwide. Computed axial tomography scan (CT scan) for this study were done at a private facility and paid for by the SISLE project, as there are no functional CT-scanners or MRI in the government health system. At facility level, patients need to pay for all services, diagnostic investigations, and treatment, however, in an effort to reduce the cost barrier for patients to access care and reduce selection bias onto the register, all investigations in our study were funded by the SISLE project whilst patients covered the cost for treatment.

### Study design and study population

The study is a case control study of PLHIV with stroke aged 18 years or older matched to HIV negative patients using the prospective observational hospital-based stroke register. We defined ‘People living with HIV’ as HIV positive patients, regardless of the time of diagnosis. From the point of being diagnosed with HIV, we considered them to fall into the category of “people living with HIV.” The term was used inclusive of anyone who has the HIV virus, regardless of how long they have had the virus or what stage of the disease they are in. This includes people who have just been diagnosed and are in the earliest stages of the infection, as well as those who have been living with the virus for many years. Stroke patients with HIV were matched by gender and age (+/− 5 years) with the HIV negative group (144) of stroke patients in a 1:4 ratio. Due to the small number of HIV patients (cases), case-control ratio was increased to 1:4 to improve representation and enable large enough sample size to conduct analysis. The Stroke register included all stroke patients aged 18 years and older between May 1, 2019, and October 8, 2021. The SISLE stroke register methods have previously been described in detail^14^.

### Clinical, laboratory and imaging data collections and definitions

Patients with suspected stroke were evaluated by a trained research team, using the National Institutes of Health Stroke Scale (NIHSS)^15^. Confirmed stroke patients were classified into sub-types by an experienced stroke physician, with reference to the clinical records and investigations; ischaemic, primary intracerebral haemorrhage, subarachnoid haemorrhage and undetermined stroke type. The Bamford classification^16^ and TOAST criteria^17,18^ were used to further sub-classify ischemic stroke. Brain neuroimaging using a CT scan was performed for 857 (87%) of patients. The Barthel ADL Index was used to measure dependency in self-care^19^

HIV test was done sequentially dependent on the availability of a testing kit, using a fourth-generation rapid diagnostic kit by SD Bioline HIV-1/2 3.0 (Standard Diagnostics Inc). The HIV testing model is provider-initiated, applying to all patients seen in the hospital. However, access to HIV testing services in this hospital is dependent on the availability of test kits and human resources. CD4 cell count was determined using the Alere Pima^™^ Analyzer (Abbott), a point-of-care testing platform with comparable performance to flow cytometry-based methods and validated in resource-limited settings^20^. CD4 laboratory test was done only for newly diagnosed HIV patients, however, this was again influenced by the availability of testing supplies.

### Data collection and analysis

All the data were collected using a standardized paper-based report form and then double entered into REDCap^™^. Univariable analyses of patients tested for HIV (511) versus those not tested (475) and confirmed stroke patients with HIV positive status (36) compared to HIV negative (475) was done. Stroke patients with HIV were matched by gender and age (+/− 5 years) with the HIV negative group (144) of stroke patients in a 1:4 ratio. The data was analyzed using IBM SPSS Statistics 27. Statistical analysis was done in contingency tables using the Pearson x^2^ for categorical variable, Paired-Test and Mann-Whitney U test for continuous variable. Adjusted Odds ratios were calculated using multi-variable log binomial models. A p-value of less than 0.05 was taken as the level of statistical significance.

### Ethical consideration

The study received ethical approval from the Sierra Leone Ethical and Scientific Review Committee on 18^th^ December 2018 and from the King’s College London (HR-18/19-8467). Written informed consent was obtained from all participants and for those unable to give consent, assent was obtained from the next of kin.

## Results

### The stroke register

**Figure 1.**
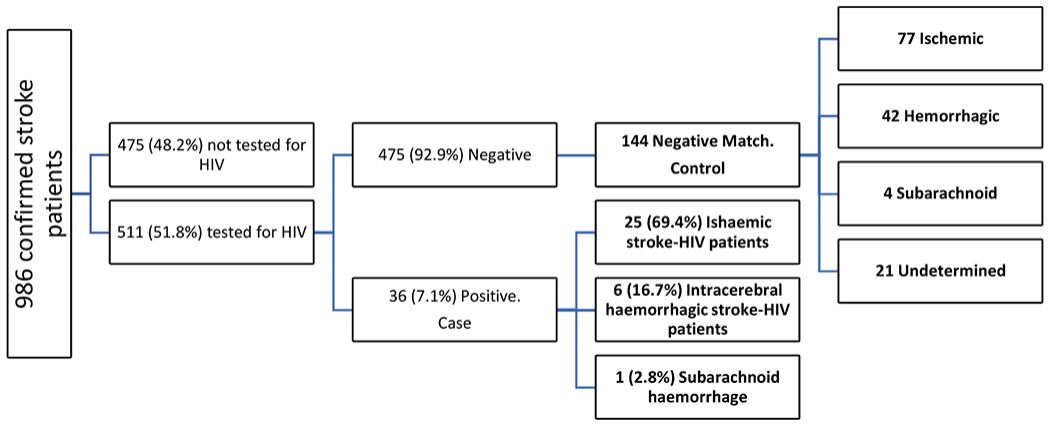
Selection process

The register recruited 986 patients with confirmed strokes, of the following stroke types, Ischaemic (625, 63.4%), Intracerebral haemorrhage (206, 20.9%), Subarachnoid haemorrhage (25, 2.5%), or undetermined (130, 13.2%) stroke types. Of these confirmed stroke cases, 511 (51.8%) were tested for HIV, of which 36 (7.1%) tested positive (Fig. 1).

### Demographic characteristics of patients with stroke

A univariable analysis of patients tested for HIV on stroke admission, including patients already known to have HIV versus those who were not tested is presented in Table I. Characteristics of patients tested vs those not tested were similar except for age (p = 0.005).

Of the 511 stroke patients who were tested for HIV, 36 were positive and 475 were negative. The mean age of PLHIV was 49 years compared with a mean age of 58 years for HIV negative patients (p = <0.001). There were more females in the HIV positive group (52.8%) compared to 49.1% in the HIV negative group (p = 0.666). Hypertension was the most prevalent risk factor in both groups but was significantly less prevalent in PLHIV 23 (63.9%) versus HIV negative 409 (86.1%) (p = 0.001). The use of any contraceptive method among females was more prevalent in PLHIV (3, 15.8%) compared HIV negative population (13, 5.6%) (p = 0.004), however, dyslipidemia was slightly more prevalent in HIV negative population (Table II).

There were no significant differences in stroke type, clinical manifestation (NIHSS), and outcome (hospital discharge and death) between PLHIV and HIV negative groups. Post-discharge follow-up using Barthel index to assess activities of daily living at 7days, 90 days, and 1 year after discharge from the hospital showed a significant improvement at 1 year in both groups (P = 0.004) compared to earlier periods (Table II & III).

Based on the demographic characteristics of PLHIV (36 confirmed stroke cases) matched with 4 HIV negative population (144 confirmed stroke cases). Due to the matching process, the mean age was similar for both PLHIV (49.1 years, standard deviation 15.1 years) and HIV negative population (49.94 years, standard deviation 14 years) (p = 0.748). The gender distribution was also similar, as slightly over 50% of patients in both groups were female (p = 0.823) (Table IV).

### Risk factors for stroke, stroke severity and stroke outcomes

Hypertension was the most prevalent risk factor for stroke in both groups, however hypertension was less frequent in the HIV negative population; 123 (85.4%) HIV negative patients and 23 (63.9%) PLHIV (p = 0.002). Prior stroke history was slightly more predominant in the HIV negative group (20, 13.9%) compared to the PLHIV (4, 11.1%) (p = 0.702) (Table IV).

Furthermore, clinical severity of stroke presentation according to NIHSS shows no-significant difference in stroke presentation among HIV negative patients (17.1, SD 9.1) compared to PLHIV (15.3, SD 7.8) (p = 0.287). The Barthel Index - 7 days post stroke which measures functional dependence outcome shows that PLHIV (25.9, SD 23.8) were not significantly different compared to HIV negative patients (34.7, SD 29.8) (p = 0.198). These comparative analyses could be limited due to the small number of HIV cases.

A higher proportion of PLHIV patients (25, 69.4%) were discharged home from the hospital when compared to HIV negative patients (95, 65.9%), and this difference was not statistically significant (p = 0.855). On the other hand, the proportion of in-hospital mortality rate was slightly lower among PLHIV (11, 30.6 %) compared to HIV negative (49, 34%) patients) (p = 0.693). However, the in-hospital mortality rate showed a significant correlation with NIHSS (22.80, SD 7.69, p = <0.001), and the severely disabled (mRS 04-05) at discharge have higher mortality at 7 days post stroke in both groups (p = <0.008) (Table V).

Of the 36 PLHIV, only 14 (38.9%) had a documented CD4+ cell count with a low mean cell count of 202.9 cell/mm^3^ (SD 342.93). CD4+ cell count testing was done only for newly diagnosed HIV patients and was influenced by the availability of testing kits. HIV-Stroke patients with CD4 + counts results (using CD4 count as a proxy for “newly diagnosed”) showed no significant change in clinical presentation severity (NIHSS) (15.9, SD 7.4, p 0.598) and mortality (61, SD 46.3, p = 0.306) (Table VI).

### Stroke subtypes and pre/post-stroke status

**Figure 2.**
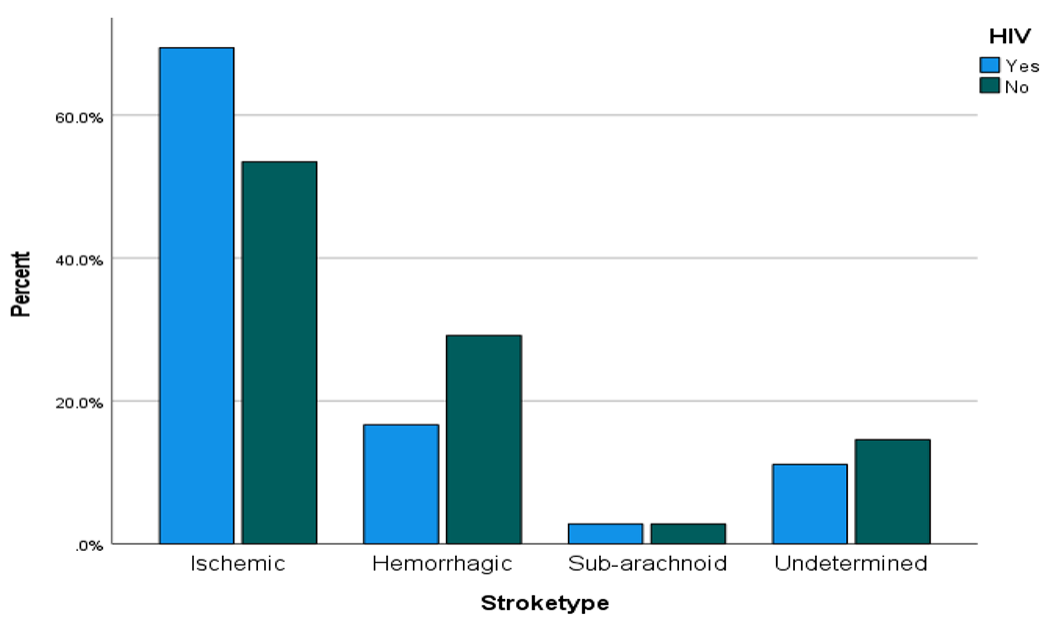
Stroke types and location of confirmed HIV positive +ve vs matched HIV negative−ve in case-control study.

Ischaemic stroke is the major stroke type reported in both groups, however, ischaemic stroke was less frequent in the HIV negative population: 77 (53.5%) compared to PLHIV:25 (69.4%) (p = 0.084). Other details on stroke type are shown in Table VII. Using the OCSP (Oxfordshire Community Stroke Project) classification; 33.3% of PLHIV have partial anterior circulation infarct (PACI) compared with 42.9%% of HIV negative population. But the frequency of some forms of ischaemic stroke is almost similar, as 28.6% of HIV negative population have lacunar cerebral infarcts, compared with 29.9% of PLHIV (Fig. 2, 3 & 4)

## Discussion

This study is the first to examine the demographics, risk factors, stroke severity (NIHSS), stroke types, and outcomes for HIV-related strokes at the main adult referral hospital in Sierra Leone. The stroke register shows that stroke occurs 10 years earlier on average in PLHIV than HIV negative population, this is similar to studies done in West Africa^7,21^. HIV testing in these facilities is not age-biased, but the availability of test kits and human resources determines access to these services. The 10-year age gap observed in stroke onset among PLHIV could be associated with HIV-related factors such as endothelial dysfunction mechanisms, which result in vasculopathy, atherosclerosis, and minor vessel diseases. These factors may be interconnected to trigger stroke onset in PLHIV^6^.

Similar to the non-HIV population, hypertension is the most prevalent risk factor for stroke in PLHIV, however significantly different between PLHIV and HIV negative population. As recent studies in Sierra Leone have shown a high burden of hypertension in PLHIV and HIV negative population, these findings, similar to a study in Cameroon, support the current calls to address comorbid NCDs in PLHIV through integrated care in low-income countries^22–24^.

Although, the evidence of HIV as a risk factor for stroke in Africa is less clear, with conflicting study results^25–28^ but in small studies of populations differing in HIV prevalence and medication take up. Ischaemic stroke is the most dominant stroke sub-type in both HIV and non-HIV stroke groups. The prevalence of ischemic stroke is similar to the SSA rate for PLHIV and HIV negative populations^29,30^. Previous studies reporting common subtypes of ischaemic strokes in both South Africa and Uganda support our findings and reinforces the fact that the management pathway for stroke and HIV should address practical challenges such as the HAART - pill access and burden, drug-drug interactions and side effects^29,30^. Furthermore, ischaemic stroke subtypes showed a different distribution in PLHIV and HIV negative population, presumably linked to the lower proportion of TACI. As this finding is linked to multiple factors, including HIV- induced endothelial dysfunction and prevailing opportunistic infections such as varicella zoster virus, especially in patients with low CD4 cell counts, it reflects on the call for early initiation of antiretroviral therapy and retention in HIV Care^31^. Furthermore, while hypertension was a most prevalent risk factor for stroke in PLHIV and HIV-negative populations, challenges with early HIV diagnosis and treatment is clearly noted in this study (Table VII), thereby underscoring our call to strengthen HIV care and reduce the risk of opportunistic infections and baseline metabolic changes in the endothelium and other tissues.

In both PLHIV and HIV-negative stroke patients, more than 50% of ischemic strokes sub-type were classified as unknown. This fact is attributable to the lack of important investigations such as carotid imaging, prolonged ECG monitoring and echocardiography in our setting. Owing to limited resources in the management of PLHIV in the country, CD4 cell count was reported for only 38.9% of PLHIV, 85% of whom have advanced HIV disease (CD4 cell count less than 200 cells/mm^3^). We add our voices for sustainable plans and funding for CD4 cell counts as it provides strategic clinical and public health information in the management of HIV in Africa^32^. Although stroke severity (NIHSS) at admission did not show significant correlation to HIV-status of patients (p = 0.147), stroke severity (NIHSS) correlated with mortality (p = <0.001). Furthermore, the 90-day mortality rate in PLHIV (41.7%) and HIV-negative (47.2%) populations reflect the health system challenges faced by these hospitals and the high mortality burden of both diseases^12,14^. Nonetheless, in PLHIV and HIV-negative stroke survivors, Barthel indices at discharge, at 90 days and at 1 year showed significant improvement.

Our study has limitations. Challenges in obtaining other investigations to confirm stroke sub-type and inability to perform HIV testing and CD4+ cell counts for a large proportion of patients significantly impact the chances of comprehensively analyzing the relationship between stroke and HIV. Also, the study was limited due to the relatively small sample size of PLHIV and HIV-related data. The use of a hospital-based register means only admitted stroke patients could be studied. Nonetheless, this study is the first to provide information on HIV-associated stroke using a prospective stroke registry from two national referral hospitals in Sierra Leone

## Conclusion

We reported stroke in HIV positive patients at the main tertiary hospital in Sierra Leone. This study shows that stroke I 10 years earlier in PLHIV than in the HIV negative population and hypertension was the most prevalent risk factor for stroke in both groups. These findings support the current call for timely diagnosis of HIV, HIV screening of stroke patients and addressing risk factors for stroke in PLHIV through integrated care.

## Figures and Tables

**Figure 3.**
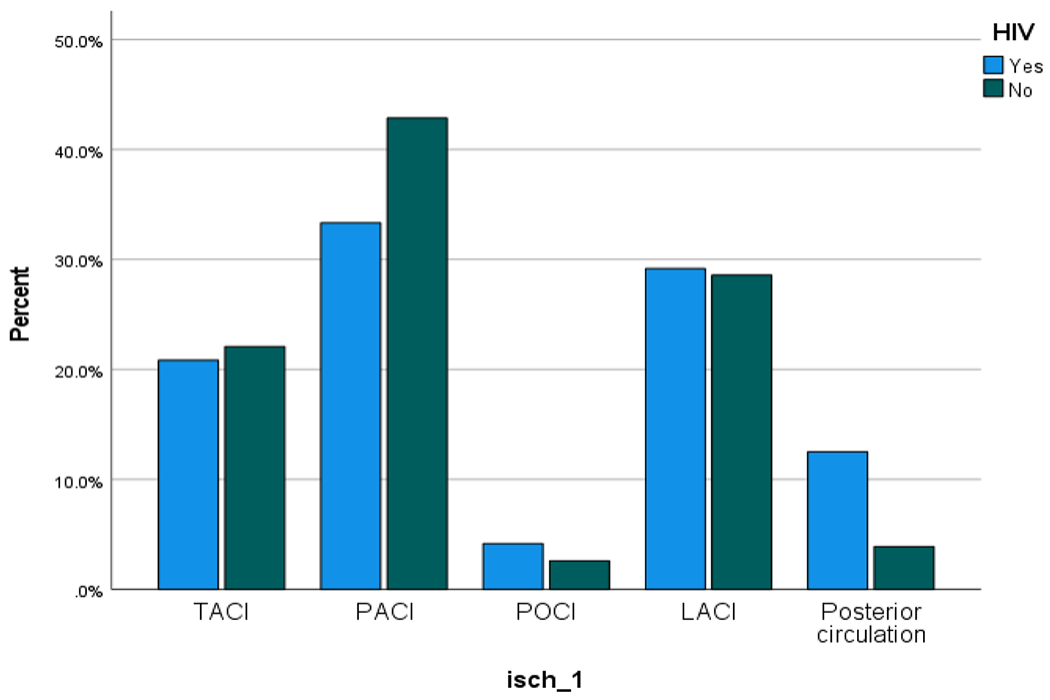
Location distribution of ischaemic stroke in confirmed HIV +ve vs matched HIV −ve in the case-control study.

**Figure 4.**
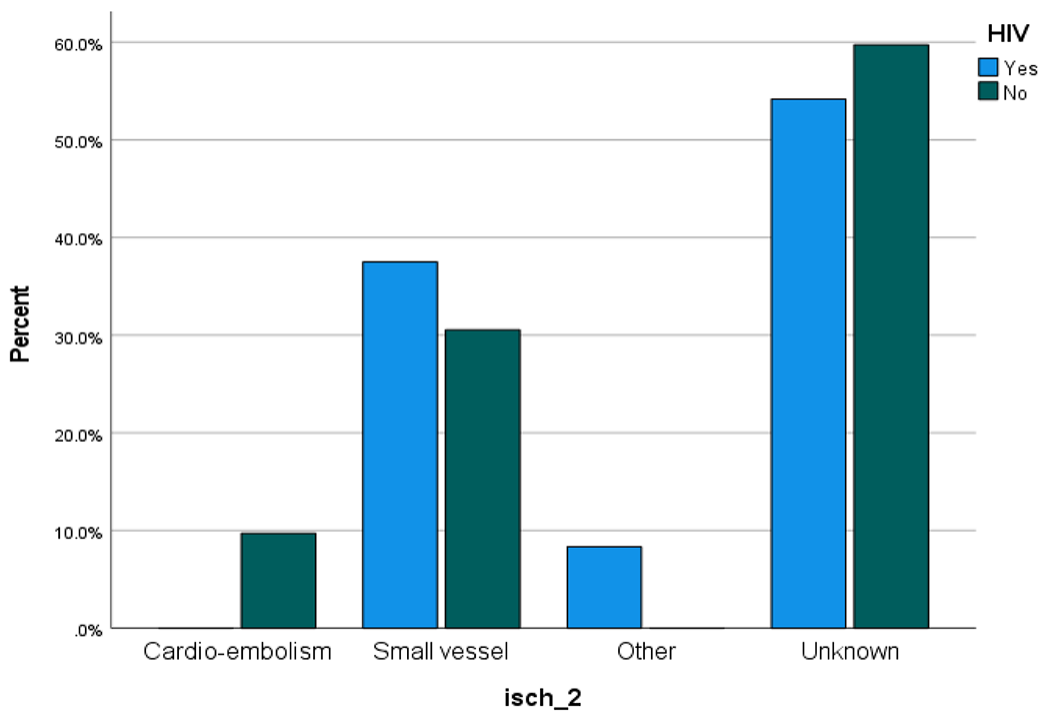
Aetiological distribution of ischaemic stroke in confirmed HIV +ve vs matched HIV −ve in the case-control group

**Table I:** Univariate analysis of tested for HIV and not tested for HIV in the stroke register, Connaught Hospital.

Features	Tested for HIV N=511	Not tested for HIV N= 475	p-value
**Age, years, mean (SD)**	**57.7 (13.9)**	**60.2 (14.0)**	<0.005*
Sex, female, n (%)	251 (49.1)	240 (50.5%)	0.659
Employment, full time, n (%)	190 (37.2)	172 (30.5%)	0.368
Prior stroke, n (%)	66 (12.9)	62 (13.1)	0.995
Diabetes mellitus, n (%)	101 (19.8)	111 (23.4)	0.307
Hypertension, n (%)	432 (84.5)	399 (84.0)	0.837
Dyslipidemia, n (%)	204 (39.9)	192 (41.6)	0.825
Smoker, n (%)	81 (15.9)	72 (15.2)	0.893
Alcohol use, n (%)	131 (225.7)	118 (26.2)	0.985
NIHSS at admission, Mean (SD)	17.04 (8.99)	16.2 (9.49)	0.152
Discharge, n (%)	319 (65.9)	311 (68.2)	0.455
Barthel Index prior to stroke, Mean (SD)	96.3 (12.7)	97.3 (11.3)	0.61
Barthel Index 7 days post stroke, Mean (SD)	25.9 (23.8)	29.5 (27.9)	0.476

**Table II:** Univariate analysis of confirmed HIV positive and HIV negative stroke patients in the stroke register at Connaught Hospital.

Features	Stroke HIV +ve N=36	Stroke HIV −ve N= 475	p-value
**Age, years, mean (SD)**	**49.08 (15.1)**	**58.31 (13.5)**	<0.001*
Sex, female, n (%)	19 (52.8)	233(49.1%)	0.666
Employment, full time, n (%)	19 (52.8)	172 (38.0%)	0.261
Education Level, No School, n (%)	9 (25.7)	208 (44.6)	0.222
Prior stroke, n (%)	4 (11.1)	61 (12.9)	0.899
Atrial Fibrillation	0	15 (3.2)	0.535
Diabetes mellitus, n (%)	4 (11.1)	97 (20.5)	0.175
**Hypertension, n (%)**	**23 (63.9)**	**409 (86.1)**	0.001*
Dyslipidemia, n (%)	12 (33.3)	192 (40.4)	0.612
Smoker, n (%)	6 (17.1)	75 (16)	0.979
Alcohol use, n (%)	12 (36.4)	118 (25.5)	0.172
**Contraceptive Use, females, n (%)**	**3 (15.8)**	**13 (5.6)**	0.004*
NIHSS at admission, Mean (SD)	15.3 (7.8)	17.2 (9.1)	0.235
Physiotherapy, n (%)	25 (71.4)	293 (62.1)	0.270
Discharge, n (%)	25 (69.4)	294 (65.5)	0.629
In hospital Death, n (%)	11 (30.6)	189 (39.8)	0.367
Dead at 90 days, n (%)	15 (41.70)	224 (47.2)	
Dead at one year, n (%)	17 (47.2)	244 (51.4)	
Death at any timepoint, n (%)	18 (50)	268 (56.4)	0.454
Barthel Index prior to stroke, Mean (SD)	96.3 (12.7)	97.3 (11.3)	0.61
Barthel Index 7 days post stroke, Mean (SD)	25.9 (23.8)	29.5 (27.9)	0.476
Barthel Index 90 days, Mean (SD)	87.3 (21.2)	75.0 (29.4)	0.07
**Barthel Index One Year, Mean (SD)**	**96.8 (9.0)**	**76.1 (25.9)**	**0.004**

**Table III:** Stroke types and OCSP (Oxfordshire Community Stroke Project) classification of patients with known HIV status in the stroke register.

Characteristics	HIV +ve	HIV −ve	p-value
Stroke type (N=36, 475) Ischaemic, n (%)	25 (69.4)	285 (60)	0.263
Haemorrhagic, n (%)	6 (16.7)	121 (25.5)	0.238
Subarachnoid, n (%)	1 (2.8)	9 (1.9)	0.712
Undetermined, n (%)	4 (11.1)	60 (12.6)	0.790
Clinical Presentation – OCSP (N=24, 282) Total Anterior Cerebral Infarct (TACI), n (%) .	5 (20.8)	55 (19.5)	0.875
Partial Anterior Cerebral Infarct (PACI), n (%) .	8 (33.3)	104 (36.9)	0.729
POCI, n (%)	1 (4.2)	15 (5.3)	0.808
Lacunar Infarct, n (%) .	7 (29.2)	95 (33.7)	0.652
Unclassified, n (%) .	3 (12.5)	13 (4.6)	0.096
Aetiology – TOAST (N= 24, 253) Large artery atherosclerosis, n (%)	0	2 (0.8)	0.662
Cardio-embolism, n (%)	0	16 (6.3)	0.204
Small vessel, n (%)	9 (37.5%)	92 (36.4)	0.912
**Other, n (%)**	**2 (8.3%)**	**1 (0.4)**	<0.001*
Unknown, n (%)	13 (54.2%)	142 (56.1)	0.853

**Table IV:** Comparison of baseline demographic and clinical features between HIV positive and matched HIV negative stroke patients in case-control study.

Features	Stroke HIV +ve N=36	Stroke HIV −ve N= 144	p-value
Age, years, Mean (SD)	49.08 (15.1)	49.94 (14.0)	0.748
Sex, female, n (%)	19 (52.8)	73(50.7)	0.823
Employment, full time, n (%)	19 (52.8)	60 (43.8)	0.762
Prior stroke, n (%)	4 (11.1)	20 (13.9)	0.702
Atrial fibrillation	0	6 (4.2%)	0.211
Diabetes mellitus, n (%)	4 (11.1)	24 (16.8)	0.402
**Hypertension, n (%)**	**23 (63.9)**	**123 (85.4)**	0.002*
Dyslipidemia, n (%)	12 (33.3)	60 (41.7)	0.481
Smoker, n (%)	6 (17.1)	25 (17.6)	0.968
Alcohol use, n (%)	12 (36.4)	36 (25.4)	0.202
Contraceptive Use, females, n (%)	3 (11.5)	7 (7.2)	0.440
NIHSS at admission, n (SD)	15.3 (7.8)	17.1 (9.1)	0.287
Discharge, n (%)	25 (69.4)	95 (65.9)	0.855
In hospital Death, n (%)	11 (30.6)	49 (34)	0.693
Barthel Index prior to stroke, Mean (SD)	96.3 (12.8)	96.7 (12.4)	0.922
Barthel Index 7 days post stroke, Mean (SD)	25.9 (23.8)	34.7 (29.8)	0.198

**Table V:** Factors associated with in-hospital patient outcome (mortality) in case-control study. Independent-samples T test.

	Mortality		p-value
	Yes = 60	No = 120	
Prior Stroke, n (%)	8 (13.6)	16 (13.3)	0.967
Hypertension, n (%)	47 (78%)	100 (83.3)	0.414
**Modified Rankin Scale 7 days post stroke, mean (SD)**	**4.13 (2.21)**	**4.84 (1.12)**	**0.008**
Diabetes, n (%)	12 (20)	16 (13.5)	0.254
**NIHSS, mean (SD)**	**22.80 (7.69)**	**13.68 (7.46)**	**<0.001**

**Table VI:** HIV-related factors on People Living HIV with stroke. Mann-Whitney U test.

		Overall	Mortality			Stroke type		
			Yes = 11	No = 25	P value	Ischaemic = 25	Haemorrhagic = 6	P value

Status of HIV diagnosis	New, n (%)		8 (72.7)	17 (68)	0.777	19 (76)	5 (83.3)	0.070
	Known, n (%)		3 (27.3)	8 (32)		6 (24)	1 (16.7)	
Anti-Retro Virals	Yes, n (%)		3 (27.3)	2 (8)	0.123	3 (12)	0	0.135
	No, n (%)		8 (72.7)	23 (92)		22 (88)	6 (100)	
	Overall N = 14	NIHSS	Mortality			Stroke type		
			Yes = 4	No = 10	P Value	Ischemic = 10	Haemorrhagic = 4	P Value
CD4+ count, Mean (SD)	202. 86 (342.9)	15.9 (7.4)	61 (46.3)	259.6 (395.8)	0.306	**94.4 (77.7)**	**474 (595.2)**	**0.057**

**Table VII:** Stroke types and OCSP classification of confirmed HIV positive vs matched HIV negative in case-control study.

Characteristics	HIV +ve	HIV −ve	p-value
Stroke type (N=36, 144)			
Ischaemic, n (%)	25 (69.4)	77 (53.5)	0.084
Haemorrhagic, n (%)	6 (16.7)	42 (29.2)	0.129
Subarachnoid, n (%)	1 (2.8)	4 (2.8)	1.000
Undetermined, n (%)	4 (11.1)	21 (14.6)	0.590
Clinical Presentation – OCSP (N=24, 77)			
Total Anterior Cerebral Infarct, n (%)	5 (20.8)	17 (22.1)	0.897
Partial Anterior Cerebral Infarct, n (%)	8 (33.3)	33 (42.9)	0.407
POCI, n (%)	1 (4.2)	2 (2.6)	0.693
Lacunar Infarct, n (%)	7 (29.2)	22 (28.6)	0.955
Unclassified, n (%)	3 (12.5)	3 (3.9)	0.119
Aetiology – TOAST (N= 24, 96)			
Cardio-embolism	0	7 (9.7%)	0.113
Small vessel	9 (37.5%)	22 (30.6%)	0.529
**Other**	**2 (8.3%)**	**0**	0.013*
Unknown	13 (54.2%)	43 (59.7%)	0.633

## Data Availability

The raw data for this study contain both personally identifiable and confidential clinical data. Requests for data access for academic use should be made to the SISLE team where data will be made available subject to academic review and acceptance of a data-sharing agreement. https://www.kcl.ac.uk/research/stroke.
